# Performance of an Artificial Intelligence Program for Assessing the Depth of Early Gastric Cancer Using Endoscopic Images

**DOI:** 10.1055/a-2957-0692

**Published:** 2026-09-17

**Authors:** Shoichiro Hirata, Kenta Hamada, Yoshiro Kawahara, Yusuke Aya, Ayano Nishio, Tomoki Yoshikawa, Yoshie Maki, Takuya Satomi, Katsunori Matsueda, Chihiro Sakaguchi, Yoshiyasu Kono, Daisuke Uchida, Masaya Iwamuro, Shinji Kuroda, Takehiro Tanaka, Motoyuki Otsuka

**Affiliations:** 1 Department of Gastroenterology and HepatologyOkayama University Graduate School of Medicine, Dentistry and Pharmaceutical SciencesOkayamaJapan; 2 Department of Practical Gastrointestinal Endoscopy, Faculty of MedicineOkayama University Graduate School of Medicine, Dentistry and Pharmaceutical SciencesOkayamaJapan; 3 Department of Regenerative Medicine, Center for Innovative Clinical Medicine 92057 Okayama University HospitalOkayamaJapan; 4 Department of Gastroenterological Surgery 199491 Okayama University Graduate School of Medicine Dentistry and Pharmaceutical SciencesOkayamaOkayamaJapan; 5 Department of PathologyOkayama University HospitalOkayamaJapan

**Keywords:** endoscopy upper GI tract, diagnosis and imaging (inc chromoendoscopy, NBI, iSCAN, FICE, CLE), precancerous conditions and cancerous lesions (dysplasia and cancer) stomach, endoscopic resection (ESD, EMRc, ...)

## Abstract

**Background and Study Aims** The depth of early gastric cancer is an important factor in determining treatment plans, such as endoscopic resection or surgery. We developed an artificial intelligence (AI) program using deep neural networks to diagnose mucosal and submucosal cancers via endoscopic images. Performance evaluation tests were conducted for its clinical applications.

**Patients and Methods** A test dataset comprising 204 early gastric cancer cases treated with endoscopic resection or surgery at our hospital between 2018 and 2021 was established. A minimum of five white-light imaging (WLI) images per case were used (median, 8; range, 5–22). The primary endpoint was the sensitivity for diagnosing mucosal cancer. The AI program was defined as useful when the lower limit of the 95% confidence interval (CI) exceeded 75%, based on previous reports on the depth diagnosis of early gastric cancer. Secondary endpoints were specificity and accuracy.

**Results** In our study, the sensitivity of this program for the diagnosis of mucosal cancer was 91.2%, and the lower limit of the 95% CI of sensitivity was 85.2%, which was higher than 75%, indicating the usefulness of this program. The specificity and accuracy were 66.2% and 82.8%, respectively.

**Conclusions** The AI program developed to diagnose the depth of early gastric cancer is useful and expected to be clinically applicable.

## Introduction

Accurate preoperative assessment of the invasion depth in early gastric cancer (EGC) is essential for selecting an optimal therapeutic strategy. Mucosal (M) cancers can be treated curatively by endoscopic submucosal dissection (ESD), whereas submucosal (SM) invasion is associated with an increased risk of lymph node metastases and generally requires surgical resection. 1 2 Consequently, misclassification of the invasion depth may lead to unnecessary surgery or noncurative endoscopic treatment, both of which have significant clinical implications.

Despite its clinical importance, the depth of diagnosis of EGC remains challenging. Conventional endoscopic assessments rely on morphologic features observed under white-light imaging (WLI), including lesion size, tumor thickness, fold convergence, surface irregularity, and marginal elevation. However, these approaches are inherently subjective and operator-dependent, resulting in limited reproducibility and suboptimal diagnostic performance, with reported accuracies typically below 80%. 3 4 Although the diagnosis of tumor depth using endoscopic ultrasonography (EUS) is advancing, there is scope for improvement. 5

Recent advances in artificial intelligence (AI), particularly deep learning-based image analysis, have enabled the development of computer-aided diagnostic systems for gastrointestinal endoscopy. Most previous studies have evaluated the overall accuracy or its performance in predicting SM invasion; however, the ability to reliably identify M cancer, which directly determines eligibility for endoscopic treatment, has received less attention. From a clinical standpoint, a high sensitivity for M cancer is particularly important to avoid unnecessary surgical intervention.

In this context, we developed a deep-learning-based AI system designed to discriminate between M and SM EGC using endoscopic images, 6 and we updated the system. The objective of this study was to evaluate the model’s performance in a real-world clinical setting using an independent follow-up cohort that was distinct from the development cohort. We predefined emphasis on the sensitivity for M cancer as the primary endpoint and demonstrated the AI-based system.

## Material and Methods

### Study Design and Setting

This retrospective study was conducted to evaluate the diagnostic performance of an AI-based system for assessing the EGC invasion depth. The AI program was developed through a collaborative effort between Okayama University Hospital and Ryobi Systems Co., Ltd. All data were collected at the Okayama University Hospital, a tertiary referral center with a high volume of endoscopic procedures.

The AI system called “Deepth EGC” was designed to classify EGC lesions as M or SM, based on endoscopic images ( Fig. 1 ). As previously reported, we used a residual network (ResNet152) during the development phase of the AI system, 6 but we have since switched to use a Vision Transformer (ViT) architecture, enabling global feature extraction by modeling the relationships between image patches. In the previous pilot study, cases were selected based on the combination of treatment modality and pathologic depth of invasion, taking into account the feasibility of patient enrollment. Consequently, M cancer cases treated by surgical resection were excluded. In contrast, the current study included gastric cancer cases with M or SM cancer who underwent either endoscopic treatment or surgical resection, including M cancer cases treated by surgical resection that had been excluded from the previous study, to create a dataset that more closely reflects real-world clinical practice.

**Fig. 1:**
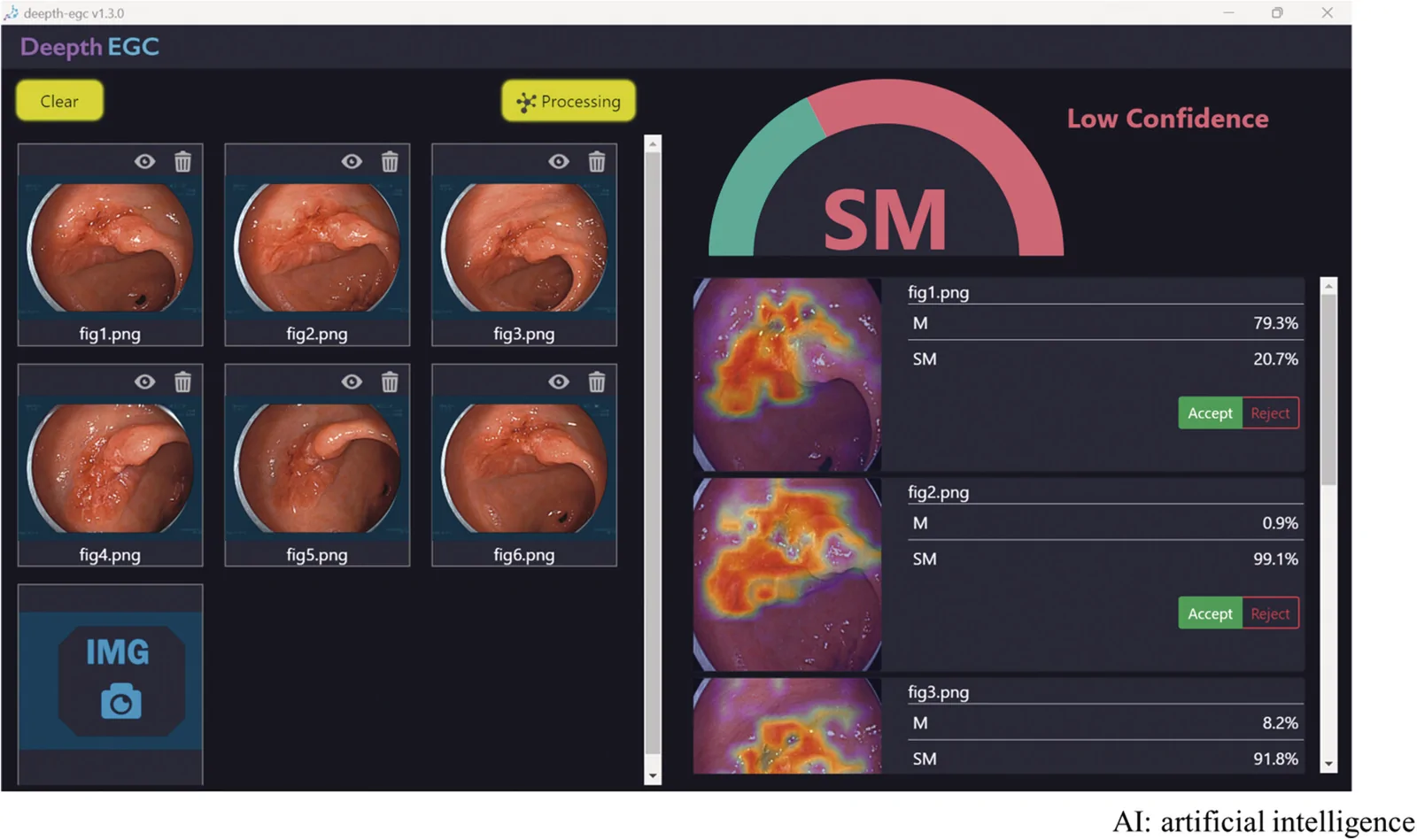
Screenshot of the AI system.

An ImageNet-pretrained ViT model was used as the backbone. During training, data augmentation was performed using random horizontal flipping and random cropping. The optimizer was stochastic gradient descent (SGD) with a momentum of 0.9, and the initial learning rate was set at 0.0001. The training batch size was 12, which was changed from that used in the previous study due to GPU memory limitations. The loss function was cross-entropy loss, and the maximum number of training epochs was set at 100. Early stopping was implemented by monitoring the validation loss, with training terminated if no improvement was observed for 10 consecutive epochs. The model from the epoch with the lowest validation loss was ultimately selected. Images with severe bleeding, motion blurring, halation, or obstruction by debris were excluded to ensure appropriate evaluation conditions. Cases were also excluded if the images did not clearly depict the lesion, if the lesion extended into the esophagus or duodenum, if the stomach had been surgically altered, or if the patient had received chemotherapy or radiotherapy within 1 year prior to endoscopy. For each lesion, multiple images were analyzed independently, and the final diagnosis was determined using a majority-voting strategy. When the number of images classified as M or SM was equal, the lesions were categorized as M. The system also generated probability scores and heat maps to visualize the regions that contributed to the predictions.

A total of 4,633 images from 368 patients were included in the dataset, with an equal number of M and SM cancer cases. The training-to-validation dataset ratio remained unchanged at 8:2. The validation performance for M cancer was as follows: sensitivity, 95.4% (95% CI, 93.5–97.0%); specificity, 84.7% (95% CI, 81.0–88.2%); and accuracy, 91.0% (95% CI, 89.1–92.8%).

### Sample Size Determination

The primary endpoint was the sensitivity of the AI system for detecting M cancer. As the sensitivity reported in previous studies ranged from 70% to 87%, we set the expected sensitivity at 85% in this study. 7 8 9 10 11 12 13 14 15 16 Assuming an expected sensitivity of 85%, the minimum required number of M cancer cases was determined to be 68, such that the lower bound of the two-sided 95% confidence interval (CI), calculated using the Wilson score method, would exceed 75%.

In addition, because the clinical role of the AI system is to assist in treatment decision-making by identifying lesions amenable to endoscopic resection, sensitivity for M cancer was prioritized over specificity in the sample-size design. To enhance the robustness of the analysis, ensure sufficient pathological variation among M cancers, and allow secondary and exploratory analyses, the target sample size was set at 136 M and 68 SM cancer cases, yielding a total of 204 cases.

### Patients and Dataset Construction

We retrospectively identified patients with histologically confirmed EGC who underwent endoscopic resection or surgical treatment between 2018 and 2021 at Okayama University Hospital. Eligible patients were adults (aged ≥20 years) with available endoscopic images suitable for depth assessment obtained using standard endoscopic systems (Olympus Corporation, Tokyo, Japan, or Fujifilm Corporation, Tokyo, Japan) with sufficient resolution and quality. Only cases with high-quality WLI endoscopic images were included, and at least five images capturing the entire tumor under adequate insufflation were required for each lesion. The same exclusion criteria as those used during system development were applied: cases with unclear images that were difficult to evaluate appropriately, cases with invasion into the esophagus or duodenum, cases involving a surgically altered stomach, and cases that had received chemotherapy or radiotherapy within the previous year. Eligible cases were selected according to these predefined criteria, resulting in a final test dataset of 204 cases comprising 136 M and 68 SM cancer cases ( Fig. 2 ).

**Fig. 2:**
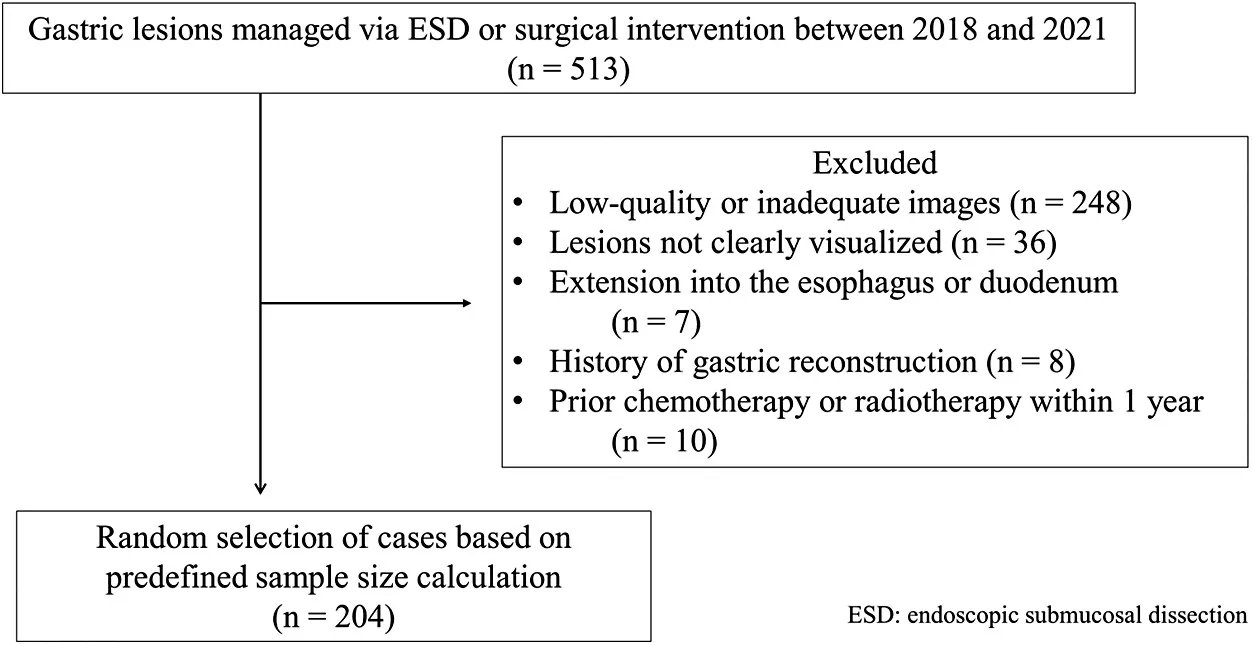
Flow diagram of the study participants.

### Image Processing, Selection, and Reference Standard

All the endoscopic images were extracted from the original data and resized to a standardized resolution. For each lesion, at least five representative images were selected by four expert endoscopists, and the selection was independently verified by additional investigators to ensure consistency and reduce selection bias.

Histopathologic diagnosis was used as the reference standard. Two experienced pathologists independently classified each lesion as M or SM cancer based on the resected specimens. Discrepancies were resolved through a consensus review. Additional clinicopathologic variables, including tumor size, location, histologic type, and ulcerative findings, were also recorded.

### Endpoints

The primary endpoint was the sensitivity of the AI system for detecting M cancer. This endpoint was selected based on its direct clinical relevance, as the accurate identification of M lesions is critical for determining eligibility for endoscopic treatment. The AI system was considered clinically useful if the lower bound of the two-sided 95% CI for sensitivity exceeded 75%, which was predefined as a clinically meaningful threshold. Previous studies have reported overall diagnostic performances of approximately 70–90% for endoscopic depth assessment of EGC, and a threshold of 75% was selected to ensure that the AI system achieved at least comparable performance while maintaining sufficiently high sensitivity for M cancer to support treatment decision-making. 7 8 9

Secondary endpoints included the specificity and overall diagnostic accuracy of the AI system for M cancer.

### Statistical Analysis

Sensitivity, specificity, and accuracy were calculated with the corresponding 95% CIs using the Wilson score method. Subgroup analyses were performed according to the endoscopic equipment and histologic characteristics. Statistical analyses were conducted using the SAS software (version 9.4; SAS Institute, Cary, NC, USA).

### Ethical Considerations

This study was approved by the Institutional Review Board of the Okayama University Hospital (protocol code: ken-2302-008. 16 December 2022). Written informed consent was obtained from the participants whenever possible; otherwise, an opt-out approach was employed in accordance with institutional guidelines. The study was conducted in compliance with the Declaration of Helsinki and relevant ethical regulations.

## Results

### Patient Characteristics

From an initial cohort of 513 patients with gastric lesions treated with ESD or surgery between 2018 and 2021, cases that met the eligibility criteria were identified. Based on a predefined sample size, 136 M cancer cases and 68 SM cancer cases were randomly selected to construct the test dataset, resulting in 204 cases ( Table 1 ). Random selection was performed to minimize selection bias and ensure that the dataset reflected the distribution of eligible cases. In total, 204 patients with EGC were included in the final analysis. The median tumor size was 14 mm (range: 2–90 mm). The lesions were distributed throughout the stomach, with the majority located in the middle and lower thirds. Most tumors were of the depressed type (0-IIc), and histologically differentiated adenocarcinoma accounted for 82% of cases. Endoscopic resection was performed in 68% of cases (139 cases), and surgical resection in 32% of cases (65 cases).

**Table 1:** The characteristics of the test dataset, *n* = 204.

Characteristic		*n* (%)
Lesion location		
	Upper third	16 (7.8)
	Middle third	106 (52)
	Lower third	82 (40)
Lesion size (mm)		
	≤ 10	65 (32)
	11–20	59 (29)
	21–50	65 (32)
	51 ≤	15 (7.3)
Macroscopic types according to the Paris Classification		
	0-I	7 (3.4)
	0-IIa	34 (17)
	0-IIb	4 (2.0)
	0-IIc	131 (64)
	0-I+IIa	1 (0.5)
	0-I+IIb	1 (0.5)
	0-I+IIc	3 (1.5)
	0-IIa+IIc	18 (8.8)
	0-IIb+IIc	3 (1.5)
	0-IIc+III	2 (1.0)
Histological type		
	Differentiated	167 (82)
	Undifferentiated	37 (18)
Ulcerative lesions		
	Absence	126 (62)
	Presence	10 (4.9)
Endoscopic systems		
	Olympus Corporation	150 (74)
	Fujifilm Corporation	54 (26)
Treatment selection		
	Endoscopic resection	139 (68)
	Surgical treatment	65 (32)

### Overall Diagnostic Performance

A confusion matrix comparing the AI-based predictions with the histopathologic diagnoses is presented in Table 2 . The AI system correctly identified 124 of the 136 M cancers and 45 of the 68 SM cancers.

**Table 2:** The confusion matrix comparing AI-based predictions with pathological diagnoses.

		Pathological diagnoses, *n*	
		pM	pSM
AI-based predictions, *n*	cM	124	23
	cSM	12	45

AI, artificial intelligence; cM, clinical mucosal lesion; cSM, clinical submucosal lesion; pM, pathologically confirmed mucosal lesion; pSM, pathologically confirmed submucosal lesion.

The sensitivity for detecting M cancer, which was the primary endpoint, was 91.2% (124/136; 95% CI, 85.2–94.9%), exceeding the predefined threshold of 75% ( Table 3 ). These results meet the criteria for clinical usefulness. The specificity for M cancer was 66.2% (45/68; 95% CI, 54.3–76.3%) and the overall diagnostic accuracy was 82.8% (169/204; 95% CI, 77.1–87.4%).

**Table 3:** The AI system’s sensitivity, specificity, and overall diagnostic accuracy for mucosal cancer.

	Estimated value, %	95% CI, %
Sensitivity	91.2	85.2–94.9
Specificity	66.2	54.3–76.3
Accuracy	82.8	77.1–87.4

AI, artificial intelligence; CI, confidence interval.

### Subgroup Analyses

The diagnostic performance of the AI system was consistent across endoscopic equipment vendors ( Table 4 ). Sensitivity for M cancer was 89.9% (95% CI, 82.4–94.4%) for Olympus systems and 94.6% (95% CI, 82.3–98.5%) for Fujifilm systems, with no statistically significant difference ( *p* = 0.51). The specificity was also comparable between the two groups (66.7% vs. 64.7%, *p* = 1.00), indicating that the model performance was not substantially affected by differences in imaging platforms.

**Table 4:** Consistency of the AI system’s diagnostic performance across endoscopic systems.

	Sensitivity		Specificity	
	Estimated value, %	95% CI, %	Estimated value, %	95% CI, %
Olympus Corporation	89.9	82.4–94.4	66.7	53.0–78.0
Fujifilm Corporation	94.6	82.3–98.5	64.7	41.3–82.7

AI, artificial intelligence; CI, confidence interval.

Table 5 shows the sensitivity of M cancers analyzed by histologic type and the presence or absence of ulcerative findings. When stratified by histologic type, the sensitivity for M cancer was higher in differentiated tumors than in undifferentiated tumors (93.2% vs. 78.9%, *p* = 0.07). Although this difference was not statistically significant, a trend toward reduced sensitivity in undifferentiated lesions was observed. The presence or absence of ulcerative findings in M cancers did not significantly influence sensitivity (90.0% vs. 91.3%, *p* = 1.00), suggesting that the AI system maintained stable performance regardless of ulcerative status.

**Table 5:** Sensitivity for mucosal cancers analyzed by histologic type and the presence or absence of ulcerative findings.

		Sensitivity	
		Estimated value, %	95% CI, %
Histological type	Differentiated	93.2	87.1–96.5
	Undifferentiated	78.9	56.7–91.5
Ulcerative findings	Absence	91.3	85.0–95.1
	Presence	90.0	59.6–98.2

AI, artificial intelligence; CI, confidence interval.

Among the 68 SM cancers, the AI system correctly classified 45 lesions (66.2%) as SM. When analyzed according to the depth of SM invasion, the correct classification rates differed between SM1 and SM2 lesions. Specifically, 10 of 20 SM1 lesions (50%) and 27 of 37 SM2 lesions (73.0%) were correctly identified as SM, indicating a tendency toward improved detection with greater invasion depth.

Collectively, these results demonstrated that the AI system achieved high sensitivity for M cancer while maintaining moderate specificity. Importantly, the diagnostic performance was robust across different endoscopic platforms and was largely unaffected by ulcerative findings, although the sensitivity appeared to be lower in undifferentiated tumors and shallow SM invasion.

## Discussion

In this study, we demonstrated that a deep learning-based AI system achieved high sensitivity (91.2%) in identifying M invasion in EGC, exceeding the predefined clinical threshold. Given that accurate identification of M lesions directly determines the eligibility for endoscopic resection, this performance supports the clinical utility of the system as a decision-support tool. Specifically, we envision that this AI system could assist in the assessment of cases with borderline indications for ESD, particularly for trainee endoscopists and clinicians at institutions with limited experience in the management of EGC.

A distinctive feature of the present study is the prioritization of sensitivity for M cancer as the primary endpoint. In clinical practice, overestimation of the invasion depth may result in unnecessary surgical resection, whereas underestimation can often be mitigated by diagnostic endoscopic resection. Therefore, a diagnostic strategy that minimizes false-negative classifications of M cancer is more aligned with current treatment paradigms. The primary clinical value of this AI system may lie in its potential to reduce the misclassification of M cancer as SM cancer, thereby helping to avoid unnecessary surgery. In particular, it may provide additional information to support decision-making regarding ESD in borderline lesions, especially in patients who are considered to be at high risk for surgical intervention. As the purpose of this study was clinical performance evaluation rather than exploratory model development, the expected sensitivity was conservatively set at 85%, which was considered a clinically meaningful performance threshold for implementation. The higher sensitivity observed in the present study compared with our previous report may be attributable, at least in part, to changes in the model architecture. However, the specific factors contributing to this difference have not yet been fully investigated.

Several studies have explored the potential of AI for predicting invasion depth in gastric cancer using endoscopic images. Previous reports have demonstrated that AI-based systems can achieve diagnostic performance comparable to that of endoscopists, with some studies suggesting further improvements when combined with human interpretation. 7 8 10 11 12 13 14 15 More recently, Lee et al. developed a deep learning model capable of predicting multiple pathologic features, including SM invasion, and reported high accuracy in internal validation; however, its performance declined substantially in external validation cohorts. 9 Similarly, Chen et al. reported decision tree and random forest-based models for depth prediction; however, they also demonstrated reduced performance on external datasets. 16 In addition, Uema et al. applied AI to EUS images and demonstrated moderate accuracy in both internal and external validation settings. 17 Collectively, these findings indicate that, although AI-based depth diagnosis is a rapidly evolving field, challenges in generalizability and clinical applicability remain.

In this context, the overall accuracy of 82.8% observed in the present study was comparable to or slightly higher than that reported in previous studies. Wu et al. reported an accuracy of approximately 78% in a prospective multicenter setting, whereas Goto et al. reported accuracies of approximately 77–78% using AI-based image analysis. 7 8 Although Lee et al. reported a higher accuracy in internal validation, their model showed a marked decline in external validation, particularly for SM invasion prediction. 9 These findings underscore the importance of evaluating AI models using clinically relevant datasets and suggest that internal performance alone may overestimate real-world applicability. Taken together, our results support the potential clinical utility of AI-assisted depth diagnosis while highlighting the need for robust validation strategies to ensure generalizability. Our approach may bridge the gap between experimental performance and real-world clinical implementations.

In contrast to prior studies that primarily focused on predicting SM invasion, the present study emphasized the detection of M cancer. This difference in endpoint selection should be considered when comparing performance across studies. Notably, no previous study has consistently demonstrated superior sensitivity for M cancer in a comparable clinical setting using WLI endoscopic images alone, suggesting that the present model demonstrates promising performance within the existing literature.

The specificity observed in the present study (66.2%) was moderate. This finding may reflect real-world clinical practice, in which lesions with uncertain depths are frequently managed with diagnostic ESD, potentially introducing a bias toward M classification. Furthermore, from a clinical perspective, lower specificity is generally less problematic than reduced sensitivity because the consequences of missing M cancer are more significant.

Subgroup analyses revealed that the model maintained a consistent performance across different endoscopic platforms, suggesting its robustness against variations in imaging systems. However, sensitivity tended to be lower in undifferentiated tumors and lesions with shallow SM invasion. These findings are consistent with the clinical experience, as such lesions often lack distinct morphologic features and remain challenging to diagnose, even for experienced endoscopists. Future model refinements incorporating more diverse datasets may improve the performance of these subgroups.

This study had several limitations. First, it was conducted at a single center, which may limit generalizability. However, this design enables a controlled evaluation of the AI system using a well-curated dataset with reliable reference standards. The present study should be interpreted as an initial step in a stepwise validation framework in which model performance was first established in a single-center setting prior to broader clinical implementation. External validation using independent datasets is essential to confirm the generalizability of the results. To address this issue, a prospective multicenter validation study is currently underway to evaluate the robustness and real-world applicability of this model. Second, the validation cohort was derived from the same institution as the development cohort. It consisted of a temporally independent patient population enrolled at a later time point. Importantly, this study was designed as a registration study with a predetermined sample size, distinguishing it from a simple retrospective post hoc analysis. Therefore, this study should be considered a clinical performance evaluation study focused on the implementation phase, which constitutes a key strength of this work. Third, the dataset’s retrospective nature may have introduced a selection bias during image acquisition. Fourth, although no significant difference was observed between endoscopy vendors, the distribution was not balanced. Fifth, one difference between this study and the previous study is that representative images were selected by specialists. During routine endoscopy, numerous images are obtained, including many that do not adequately depict the lesion. For the analysis, images that did not depict the lesion or were considered unsuitable for diagnosis were excluded, and only images appropriate for lesion evaluation were used. This image-selection process was performed in the same manner in both the previous and the current studies. Therefore, we believe that the specialist selection of representative images in the current study did not substantially affect the sensitivity for M cancer. Finally, the model was evaluated using still images rather than real-time videos, which may limit its direct applicability in clinical practice.

Future research should focus on prospective multicenter validation and real-time implementation of AI systems in endoscopic workflows. In addition, integrating AI predictions with endoscopist interpretations may further enhance diagnostic performance, as suggested in previous studies. 8 The incorporation of multimodal data, including magnifying endoscopy or EUS, may also improve the accuracy of depth assessment. Notably, the AI system evaluated in this study has recently been approved as a medical device under the Pharmaceuticals and Medical Devices Act in Japan and is commercially available as a clinical decision-support tool. Importantly, the system is intended to assist, rather than replace, endoscopist judgment, and further post-marketing validation in routine clinical practice is essential. Determining whether AI-assisted approaches improve diagnostic accuracy is an important issue that warrants further investigation and will be addressed in future prospective studies.

In conclusion, the AI-based system demonstrated high sensitivity for the detection of M cancer and robust diagnostic performance. Our findings provide clinically relevant evidence supporting the integration of AI into routine endoscopic decision-making with the potential to optimize treatment selection and minimize unnecessary surgical intervention in EGC.
